# Tetra-μ-benzoato-bis­[(3,5-dimethyl­pyridine)­copper(II)]

**DOI:** 10.1107/S1600536812008604

**Published:** 2012-03-17

**Authors:** Qian Guo, Ping Wang, Fu-Chen Liu

**Affiliations:** a School of Chemistry and Chemical Engineering, Tianjin University of Technology, Tianjin 300191, People’s Republic of China

## Abstract

In the centrosymmetric binuclear title compound, [Cu_2_(C_7_H_5_O_2_)_4_(C_7_H_9_N)_2_], the Cu^II^ atom is coordinated by four O atoms from benzoate anions and one N atom from a dimethyl­pyridine ligand. A paddle-wheel-like dimer is formed by two Cu^II^ ions and four benzoate anions with two 3,5-dimethyl­pyridine ligands at the axial position of the Cu^II^ ions. The dihedral angle between the two unique benzene rings is 84.26 (16)°. The dihedral angles between the pyridine ring and the benzene rings are 61.67 (15) and 34.27 (14)°. There is π–π stacking of inversion-related pyridine rings, with a centroid–centroid distance of 3.833 (2) Å.

## Related literature

For a general review of copper(II) carboxyl­ates, see: Doedens (1976 ▶). For the crystal structures of similar complexes, see: Speier & Fulop (1989 ▶).
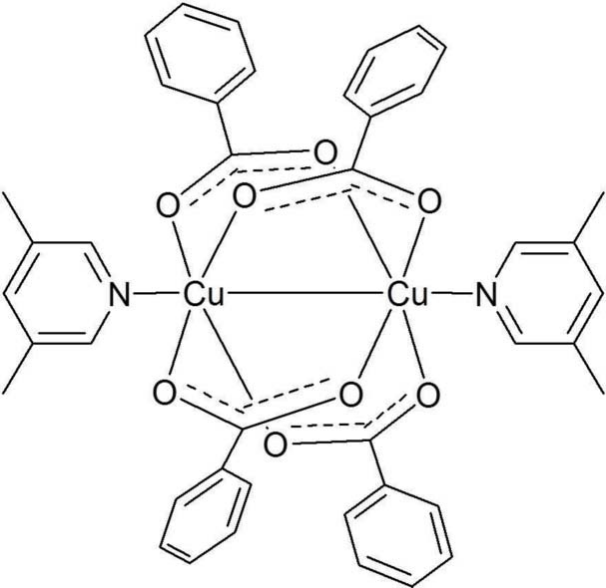


## Experimental

### 

#### Crystal data

[Cu_2_(C_7_H_5_O_2_)_4_(C_7_H_9_N)_2_]
*M* *_r_* = 825.84
Triclinic, 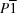

*a* = 10.249 (2) Å
*b* = 10.619 (2) Å
*c* = 10.752 (2) Å
α = 64.14 (3)°
β = 67.34 (3)°
γ = 80.36 (3)°
*V* = 971.7 (5) Å^3^
*Z* = 1
Mo *K*α radiation
μ = 1.15 mm^−1^
*T* = 293 K
0.20 × 0.18 × 0.18 mm

#### Data collection

Rigaku SCXmini diffractometer
Absorption correction: multi-scan (*ABSCOR*; Higashi, 1995 ▶) *T*_min_ = 0.461, *T*_max_ = 1
10126 measured reflections
4417 independent reflections
2943 reflections with *I* > 2σ(*I*)
*R*_int_ = 0.067

#### Refinement

*R*[*F* ^2^ > 2σ(*F* ^2^)] = 0.067
*wR*(*F* ^2^) = 0.162
*S* = 1.06
4417 reflections
244 parameters
H-atom parameters constrained
Δρ_max_ = 0.46 e Å^−3^
Δρ_min_ = −0.40 e Å^−3^

### 

Data collection: *PROCESS-AUTO* (Rigaku, 1998 ▶); cell refinement: *PROCESS-AUTO*; data reduction: *PROCESS-AUTO*; program(s) used to solve structure: *SHELXS97* (Sheldrick, 2008 ▶); program(s) used to refine structure: *SHELXL97* (Sheldrick, 2008 ▶); molecular graphics: *XP* in *SHELXTL* (Sheldrick, 2008 ▶); software used to prepare material for publication: *SHELXTL*.

## Supplementary Material

Crystal structure: contains datablock(s) global, I. DOI: 10.1107/S1600536812008604/pk2382sup1.cif

Structure factors: contains datablock(s) I. DOI: 10.1107/S1600536812008604/pk2382Isup2.hkl

Additional supplementary materials:  crystallographic information; 3D view; checkCIF report

## Figures and Tables

**Table 1:** Selected bond lengths (Å)

Cu1—O2	1.953 (3)
Cu1—O1^i^	1.966 (3)
Cu1—O4^i^	1.968 (3)
Cu1—O3	1.969 (3)
Cu1—N1	2.182 (3)
Cu1—Cu1^i^	2.6721 (13)

Symmetry code: (i) 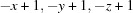.

**Fig. 1.**
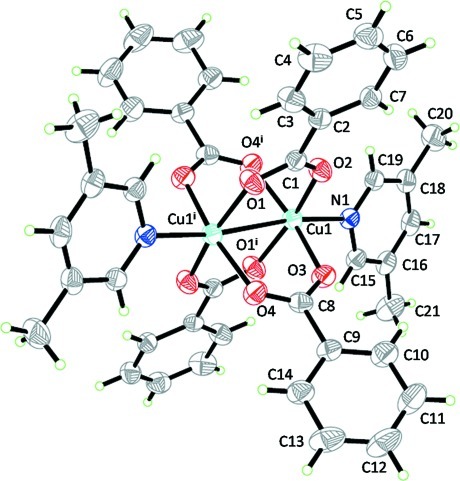
The molecular structure of the title compound. Ellipsoids are drawn at the 30% probability level. Only the asymmetric unit is labeled. Symmetry code: i = -x+1, -y+1, -z+1.

## supplementary crystallographic information

### Comment

The binuclear paddle-wheel cage structure of copper(II) carboxylates is well established (Doedens, 1976; Speier & Fulop, 1989). Here we report the synthesis and crystal structure of a new copper complex with 3,5-dimethylpyridine and benzoic acid ligands. Each Cu^II^ is coordinated by one 3,5-dimethylpyridine ligand and two benzoate ligands. A pair of Cu^II^ ions are connected through four *syn*-*syn* bidentate chelating carboxylate bridges to generate a paddle wheel binuclear unit (Fig. 1). There is π-π stacking of inversion related pyridine rings related by symmetry operation: 1-x,-y,2-z; and the centroid-centroid distances is 3.833 (2)Å.

### Experimental

A mixture of Cu(II) chloride (2 mmol), benzoic acid (1mmol) and 3,5-dimethylpyridine (0.5mmol), in 10 ml aqueous solution was sealed in a teflon-lined stainless-steel Parr bomb that was heated at 413 K for 48 h. Green crystals of the title complex were collected after the bomb was allowed to cool to room temperature. Yield 20% based on metal salt.

### Refinement

All hydrogen atoms were included in calculated positions and treated as riding on their parent C atoms with C—H = 0.93Å and Uiso(H) = 1.2Ueq(C), or 0.96Å and Uiso = 1.5Ueq(C) for the methyl H atoms.

### Crystal data

**Table:**

[Cu_2_(C_7_H_5_O_2_)_4_(C_7_H_9_N)_2_]	*Z* = 1
*M**_r_* = 825.84	*F*(000) = 426
Triclinic, *P*1	*D*_x_ = 1.411 Mg m^−^^3^
Hall symbol: -P 1	Mo *K*α radiation, λ = 0.71073 Å
*a* = 10.249 (2) Å	Cell parameters from 8517 reflections
*b* = 10.619 (2) Å	θ = 3.0–27.9°
*c* = 10.752 (2) Å	µ = 1.15 mm^−^^1^
α = 64.14 (3)°	*T* = 293 K
β = 67.34 (3)°	Block, green
γ = 80.36 (3)°	0.2 × 0.18 × 0.18 mm
*V* = 971.7 (5) Å^3^	

### Data collection

**Table:**

Rigaku SCXmini diffractometer	4417 independent reflections
Radiation source: fine-focus sealed tube	2943 reflections with *I* > 2σ(*I*)
Graphite monochromator	*R*_int_ = 0.067
ω scans	θ_max_ = 27.5°, θ_min_ = 3.0°
Absorption correction: multi-scan (*ABSCOR*; Higashi, 1995)	*h* = −13→13
*T*_min_ = 0.461, *T*_max_ = 1	*k* = −13→13
10126 measured reflections	*l* = −13→13

### Refinement

**Table:**

Refinement on *F*^2^	Primary atom site location: structure-invariant direct methods
Least-squares matrix: full	Secondary atom site location: difference Fourier map
*R*[*F*^2^ > 2σ(*F*^2^)] = 0.067	Hydrogen site location: inferred from neighbouring sites
*wR*(*F*^2^) = 0.162	H-atom parameters constrained
*S* = 1.06	*w* = 1/[σ^2^(*F*_o_^2^) + (0.0637*P*)^2^] where *P* = (*F*_o_^2^ + 2*F*_c_^2^)/3
4417 reflections	(Δ/σ)_max_ < 0.001
244 parameters	Δρ_max_ = 0.46 e Å^−^^3^
0 restraints	Δρ_min_ = −0.40 e Å^−^^3^

### Special details

**Table:**

Geometry. All esds (except the esd in the dihedral angle between two l.s. planes) are estimated using the full covariance matrix. The cell esds are taken into account individually in the estimation of esds in distances, angles and torsion angles; correlations between esds in cell parameters are only used when they are defined by crystal symmetry. An approximate (isotropic) treatment of cell esds is used for estimating esds involving l.s. planes.
Refinement. Refinement of F^2^ against ALL reflections. The weighted R-factor wR and goodness of fit S are based on F^2^, conventional R-factors R are based on F, with F set to zero for negative F^2^. The threshold expression of F^2^ > 2σ(F^2^) is used only for calculating R-factors(gt) etc. and is not relevant to the choice of reflections for refinement. R-factors based on F^2^ are statistically about twice as large as those based on F, and R- factors based on ALL data will be even larger.

### Fractional atomic coordinates and isotropic or equivalent isotropic displacement parameters (Å^2^)

**Table:**

	*x*	*y*	*z*	*U*_iso_*/*U*_eq_	
O1	0.3897 (3)	0.3033 (3)	0.5902 (3)	0.0637 (8)	
Cu1	0.44953 (5)	0.61486 (4)	0.41389 (5)	0.0490 (2)	
O3	0.3341 (3)	0.6269 (3)	0.6031 (3)	0.0608 (8)	
O4	0.4237 (3)	0.4387 (3)	0.7466 (3)	0.0618 (8)	
O2	0.3048 (3)	0.4950 (3)	0.4447 (3)	0.0609 (8)	
N1	0.3873 (3)	0.8205 (3)	0.2789 (3)	0.0495 (8)	
C1	0.3029 (4)	0.3663 (4)	0.5251 (4)	0.0490 (10)	
C4	0.0875 (6)	0.0579 (5)	0.6323 (5)	0.0804 (15)	
H4A	0.0884	−0.0391	0.6813	0.096*	
C8	0.3451 (4)	0.5443 (4)	0.7258 (4)	0.0535 (10)	
C2	0.1904 (4)	0.2791 (4)	0.5442 (4)	0.0492 (9)	
C3	0.1912 (5)	0.1358 (5)	0.6164 (5)	0.0663 (12)	
H3A	0.2623	0.0917	0.6544	0.080*	
C16	0.3891 (5)	1.0667 (4)	0.2119 (5)	0.0617 (11)	
C7	0.0844 (4)	0.3430 (5)	0.4877 (4)	0.0551 (10)	
H7A	0.0834	0.4398	0.4379	0.066*	
C9	0.2579 (5)	0.5741 (4)	0.8573 (4)	0.0559 (11)	
C10	0.1364 (5)	0.6526 (5)	0.8600 (5)	0.0776 (14)	
H10A	0.1093	0.6898	0.7777	0.093*	
C15	0.4131 (4)	0.9293 (4)	0.2955 (4)	0.0563 (11)	
H15A	0.4498	0.9119	0.3683	0.068*	
C17	0.3314 (5)	1.0881 (5)	0.1066 (5)	0.0667 (13)	
H17A	0.3126	1.1787	0.0477	0.080*	
C19	0.3335 (4)	0.8451 (4)	0.1775 (4)	0.0533 (10)	
H19A	0.3157	0.7691	0.1650	0.064*	
C13	0.2201 (7)	0.5513 (6)	1.0997 (5)	0.0948 (18)	
H13A	0.2496	0.5188	1.1801	0.114*	
C6	−0.0199 (5)	0.2633 (6)	0.5051 (5)	0.0703 (13)	
H6A	−0.0917	0.3066	0.4680	0.084*	
C18	0.3019 (4)	0.9778 (5)	0.0880 (4)	0.0613 (12)	
C11	0.0548 (6)	0.6765 (6)	0.9830 (6)	0.0997 (19)	
H11A	−0.0297	0.7256	0.9857	0.120*	
C20	0.2345 (6)	0.9980 (6)	−0.0211 (5)	0.0943 (17)	
H20A	0.2206	1.0961	−0.0731	0.141*	
H20B	0.1448	0.9509	0.0303	0.141*	
H20C	0.2949	0.9600	−0.0901	0.141*	
C14	0.2992 (5)	0.5227 (5)	0.9776 (5)	0.0695 (13)	
H14A	0.3806	0.4685	0.9773	0.083*	
C21	0.4241 (6)	1.1845 (5)	0.2354 (6)	0.0987 (19)	
H21A	0.4002	1.2720	0.1680	0.148*	
H21B	0.5234	1.1830	0.2182	0.148*	
H21C	0.3711	1.1738	0.3350	0.148*	
C5	−0.0179 (5)	0.1220 (6)	0.5762 (5)	0.0759 (14)	
H5A	−0.0878	0.0683	0.5871	0.091*	
C12	0.0977 (7)	0.6282 (6)	1.1015 (6)	0.102 (2)	
H12A	0.0441	0.6472	1.1834	0.123*	

### Atomic displacement parameters (Å^2^)

**Table:**

	*U*^11^	*U*^22^	*U*^33^	*U*^12^	*U*^13^	*U*^23^
O1	0.074 (2)	0.0512 (16)	0.069 (2)	−0.0033 (15)	−0.0414 (17)	−0.0118 (15)
Cu1	0.0615 (4)	0.0408 (3)	0.0417 (3)	0.0041 (2)	−0.0241 (2)	−0.0106 (2)
O3	0.079 (2)	0.0576 (17)	0.0393 (15)	0.0088 (15)	−0.0218 (14)	−0.0163 (14)
O4	0.078 (2)	0.0530 (17)	0.0469 (16)	0.0144 (16)	−0.0243 (15)	−0.0171 (14)
O2	0.071 (2)	0.0466 (17)	0.0672 (19)	0.0010 (14)	−0.0382 (16)	−0.0133 (14)
N1	0.059 (2)	0.0409 (18)	0.0423 (18)	0.0047 (16)	−0.0183 (16)	−0.0123 (15)
C1	0.056 (3)	0.048 (2)	0.042 (2)	0.006 (2)	−0.0188 (19)	−0.0188 (19)
C4	0.108 (4)	0.052 (3)	0.072 (3)	−0.022 (3)	−0.029 (3)	−0.012 (2)
C8	0.067 (3)	0.049 (2)	0.042 (2)	−0.006 (2)	−0.020 (2)	−0.015 (2)
C2	0.055 (3)	0.057 (2)	0.035 (2)	0.001 (2)	−0.0134 (18)	−0.0199 (18)
C3	0.083 (3)	0.054 (3)	0.061 (3)	−0.007 (2)	−0.036 (2)	−0.012 (2)
C16	0.064 (3)	0.043 (2)	0.058 (3)	0.003 (2)	−0.013 (2)	−0.011 (2)
C7	0.051 (3)	0.062 (3)	0.052 (2)	0.005 (2)	−0.016 (2)	−0.026 (2)
C9	0.073 (3)	0.046 (2)	0.045 (2)	−0.002 (2)	−0.019 (2)	−0.0167 (19)
C10	0.100 (4)	0.069 (3)	0.059 (3)	0.014 (3)	−0.030 (3)	−0.025 (3)
C15	0.065 (3)	0.050 (2)	0.048 (2)	0.008 (2)	−0.021 (2)	−0.016 (2)
C17	0.066 (3)	0.055 (3)	0.048 (3)	0.015 (2)	−0.015 (2)	−0.004 (2)
C19	0.056 (3)	0.057 (3)	0.042 (2)	0.008 (2)	−0.0171 (19)	−0.019 (2)
C13	0.144 (6)	0.082 (4)	0.049 (3)	−0.004 (4)	−0.025 (3)	−0.025 (3)
C6	0.054 (3)	0.093 (4)	0.071 (3)	0.006 (3)	−0.023 (2)	−0.041 (3)
C18	0.060 (3)	0.067 (3)	0.039 (2)	0.010 (2)	−0.018 (2)	−0.010 (2)
C11	0.110 (5)	0.092 (4)	0.083 (4)	0.031 (4)	−0.021 (4)	−0.046 (3)
C20	0.108 (4)	0.109 (4)	0.064 (3)	0.025 (3)	−0.050 (3)	−0.025 (3)
C14	0.092 (4)	0.063 (3)	0.050 (3)	0.005 (3)	−0.026 (2)	−0.021 (2)
C21	0.133 (5)	0.051 (3)	0.094 (4)	−0.003 (3)	−0.027 (4)	−0.024 (3)
C5	0.068 (3)	0.089 (4)	0.075 (3)	−0.011 (3)	−0.017 (3)	−0.041 (3)
C12	0.141 (6)	0.087 (4)	0.056 (3)	0.016 (4)	−0.011 (3)	−0.034 (3)

### Geometric parameters (Å, º)

**Table:**

O1—C1	1.265 (4)	C9—C14	1.371 (6)
O1—Cu1^i^	1.966 (3)	C9—C10	1.375 (6)
Cu1—O2	1.953 (3)	C10—C11	1.372 (6)
Cu1—O1^i^	1.966 (3)	C10—H10A	0.9300
Cu1—O4^i^	1.968 (3)	C15—H15A	0.9300
Cu1—O3	1.969 (3)	C17—C18	1.368 (6)
Cu1—N1	2.182 (3)	C17—H17A	0.9300
Cu1—Cu1^i^	2.6721 (13)	C19—C18	1.386 (5)
O3—C8	1.263 (4)	C19—H19A	0.9300
O4—C8	1.254 (4)	C13—C12	1.376 (7)
O4—Cu1^i^	1.968 (3)	C13—C14	1.385 (6)
O2—C1	1.258 (4)	C13—H13A	0.9300
N1—C19	1.315 (5)	C6—C5	1.356 (6)
N1—C15	1.325 (5)	C6—H6A	0.9300
C1—C2	1.496 (5)	C18—C20	1.502 (6)
C4—C3	1.369 (6)	C11—C12	1.366 (7)
C4—C5	1.375 (7)	C11—H11A	0.9300
C4—H4A	0.9300	C20—H20A	0.9600
C8—C9	1.490 (5)	C20—H20B	0.9600
C2—C3	1.374 (5)	C20—H20C	0.9600
C2—C7	1.383 (5)	C14—H14A	0.9300
C3—H3A	0.9300	C21—H21A	0.9600
C16—C15	1.380 (5)	C21—H21B	0.9600
C16—C17	1.390 (6)	C21—H21C	0.9600
C16—C21	1.501 (6)	C5—H5A	0.9300
C7—C6	1.380 (6)	C12—H12A	0.9300
C7—H7A	0.9300		
			
C1—O1—Cu1^i^	127.3 (3)	C11—C10—C9	120.7 (5)
O2—Cu1—O1^i^	167.39 (11)	C11—C10—H10A	119.7
O2—Cu1—O4^i^	88.23 (13)	C9—C10—H10A	119.7
O1^i^—Cu1—O4^i^	90.12 (13)	N1—C15—C16	124.3 (4)
O2—Cu1—O3	89.09 (13)	N1—C15—H15A	117.8
O1^i^—Cu1—O3	89.74 (13)	C16—C15—H15A	117.8
O4^i^—Cu1—O3	167.12 (11)	C18—C17—C16	121.0 (4)
O2—Cu1—N1	101.52 (12)	C18—C17—H17A	119.5
O1^i^—Cu1—N1	91.10 (12)	C16—C17—H17A	119.5
O4^i^—Cu1—N1	98.25 (12)	N1—C19—C18	123.8 (4)
O3—Cu1—N1	94.63 (12)	N1—C19—H19A	118.1
O2—Cu1—Cu1^i^	87.13 (8)	C18—C19—H19A	118.1
O1^i^—Cu1—Cu1^i^	80.26 (9)	C12—C13—C14	119.7 (5)
O4^i^—Cu1—Cu1^i^	84.30 (8)	C12—C13—H13A	120.1
O3—Cu1—Cu1^i^	82.99 (8)	C14—C13—H13A	120.1
N1—Cu1—Cu1^i^	171.02 (9)	C5—C6—C7	120.1 (4)
C8—O3—Cu1	124.2 (3)	C5—C6—H6A	120.0
C8—O4—Cu1^i^	122.9 (2)	C7—C6—H6A	120.0
C1—O2—Cu1	119.9 (3)	C17—C18—C19	117.0 (4)
C19—N1—C15	117.8 (3)	C17—C18—C20	121.9 (4)
C19—N1—Cu1	125.0 (3)	C19—C18—C20	121.0 (4)
C15—N1—Cu1	117.1 (3)	C12—C11—C10	119.9 (5)
O2—C1—O1	125.4 (4)	C12—C11—H11A	120.0
O2—C1—C2	117.8 (3)	C10—C11—H11A	120.0
O1—C1—C2	116.8 (3)	C18—C20—H20A	109.5
C3—C4—C5	120.5 (5)	C18—C20—H20B	109.5
C3—C4—H4A	119.8	H20A—C20—H20B	109.5
C5—C4—H4A	119.8	C18—C20—H20C	109.5
O4—C8—O3	125.5 (4)	H20A—C20—H20C	109.5
O4—C8—C9	116.9 (3)	H20B—C20—H20C	109.5
O3—C8—C9	117.6 (4)	C9—C14—C13	120.1 (5)
C3—C2—C7	119.3 (4)	C9—C14—H14A	119.9
C3—C2—C1	121.0 (4)	C13—C14—H14A	119.9
C7—C2—C1	119.7 (4)	C16—C21—H21A	109.5
C4—C3—C2	120.0 (4)	C16—C21—H21B	109.5
C4—C3—H3A	120.0	H21A—C21—H21B	109.5
C2—C3—H3A	120.0	C16—C21—H21C	109.5
C15—C16—C17	116.1 (4)	H21A—C21—H21C	109.5
C15—C16—C21	121.1 (4)	H21B—C21—H21C	109.5
C17—C16—C21	122.9 (4)	C6—C5—C4	120.0 (5)
C6—C7—C2	120.1 (4)	C6—C5—H5A	120.0
C6—C7—H7A	119.9	C4—C5—H5A	120.0
C2—C7—H7A	119.9	C11—C12—C13	120.1 (5)
C14—C9—C10	119.3 (4)	C11—C12—H12A	120.0
C14—C9—C8	119.6 (4)	C13—C12—H12A	120.0
C10—C9—C8	121.1 (4)		
			
O2—Cu1—O3—C8	91.1 (3)	C5—C4—C3—C2	0.1 (7)
O1^i^—Cu1—O3—C8	−76.3 (3)	C7—C2—C3—C4	−0.3 (6)
O4^i^—Cu1—O3—C8	13.1 (7)	C1—C2—C3—C4	−179.8 (4)
N1—Cu1—O3—C8	−167.4 (3)	C3—C2—C7—C6	0.6 (6)
Cu1^i^—Cu1—O3—C8	3.9 (3)	C1—C2—C7—C6	−179.9 (3)
O1^i^—Cu1—O2—C1	2.3 (7)	O4—C8—C9—C14	23.0 (6)
O4^i^—Cu1—O2—C1	85.0 (3)	O3—C8—C9—C14	−156.8 (4)
O3—Cu1—O2—C1	−82.4 (3)	O4—C8—C9—C10	−157.0 (4)
N1—Cu1—O2—C1	−176.9 (3)	O3—C8—C9—C10	23.1 (6)
Cu1^i^—Cu1—O2—C1	0.6 (3)	C14—C9—C10—C11	−1.8 (7)
O2—Cu1—N1—C19	−36.0 (3)	C8—C9—C10—C11	178.3 (4)
O1^i^—Cu1—N1—C19	144.2 (3)	C19—N1—C15—C16	−1.0 (6)
O4^i^—Cu1—N1—C19	53.9 (3)	Cu1—N1—C15—C16	175.5 (3)
O3—Cu1—N1—C19	−126.0 (3)	C17—C16—C15—N1	1.4 (7)
Cu1^i^—Cu1—N1—C19	159.9 (4)	C21—C16—C15—N1	−178.7 (4)
O2—Cu1—N1—C15	147.9 (3)	C15—C16—C17—C18	−0.4 (7)
O1^i^—Cu1—N1—C15	−32.0 (3)	C21—C16—C17—C18	179.6 (4)
O4^i^—Cu1—N1—C15	−122.3 (3)	C15—N1—C19—C18	−0.3 (6)
O3—Cu1—N1—C15	57.8 (3)	Cu1—N1—C19—C18	−176.5 (3)
Cu1^i^—Cu1—N1—C15	−16.3 (8)	C2—C7—C6—C5	−0.8 (6)
Cu1—O2—C1—O1	−0.6 (6)	C16—C17—C18—C19	−0.7 (7)
Cu1—O2—C1—C2	−179.6 (2)	C16—C17—C18—C20	177.6 (4)
Cu1^i^—O1—C1—O2	0.1 (6)	N1—C19—C18—C17	1.1 (7)
Cu1^i^—O1—C1—C2	179.1 (2)	N1—C19—C18—C20	−177.2 (4)
Cu1^i^—O4—C8—O3	1.6 (6)	C9—C10—C11—C12	3.3 (8)
Cu1^i^—O4—C8—C9	−178.3 (3)	C10—C9—C14—C13	−0.8 (7)
Cu1—O3—C8—O4	−4.5 (6)	C8—C9—C14—C13	179.1 (4)
Cu1—O3—C8—C9	175.3 (3)	C12—C13—C14—C9	1.8 (8)
O2—C1—C2—C3	172.5 (4)	C7—C6—C5—C4	0.6 (7)
O1—C1—C2—C3	−6.5 (6)	C3—C4—C5—C6	−0.3 (8)
O2—C1—C2—C7	−7.0 (5)	C10—C11—C12—C13	−2.3 (9)
O1—C1—C2—C7	174.0 (3)	C14—C13—C12—C11	−0.3 (9)

Symmetry code: (i) −*x*+1, −*y*+1, −*z*+1.
